# Long-term follow-up of females with unbalanced X;Y translocations—reproductive and nonreproductive consequences

**DOI:** 10.1186/s13039-015-0112-0

**Published:** 2015-02-22

**Authors:** Whitney A Dobek, Hyung-Goo Kim, Cedric A Walls, Lynn P Chorich, Sandra PT Tho, Zi-Xuan Wang, Paul G McDonough, Lawrence C Layman

**Affiliations:** Department of Obstetrics & Gynecology, Medical College of Georgia, Georgia Regents University, Augusta, GA USA; Section of Reproductive Endocrinology, Infertility, & Genetics, Medical College of Georgia, Georgia Regents University, Augusta, GA USA; Department of Surgery and Department of Pathology, Anatomy & Cell Biology, Thomas Jefferson University, Philadelphia, PA USA; Section of Reproductive Endocrinology, Infertility, & Genetics, Department of Obstetrics & Gynecology, Medical College of Georgia, Georgia Regents University, 1120 15th Street, Augusta, GA USA

**Keywords:** X;Y translocation, Xp22 deletion, Chromosome translocation, Short stature, SHOX gene, Kallmann syndrome, KAL1 gene

## Abstract

**Background:**

Females with Xp;Yq translocations manifest short stature and normal fertility, but rarely have follow-up. The study purpose was to define the phenotype of a family with t(X;Y)(p22.3;q11.2), determine long-term reproductive function, and compare to all reported female cases.

**Methods:**

Comprehensive clinical and molecular analyses were performed on the female proband, who had regular menses, normal endocrine function, and three pregnancies spanning seven years--a normal liveborn male and two with unbalanced translocations (liveborn female and stillborn male).

**Results:**

The translocation truncated *KAL1* and deleted 44 genes on der(X). Our report constitutes the longest follow-up of an X;Y translocation female. She had no evidence of Kallmann syndrome, gonadoblastoma, or cardiovascular disease. Detailed analysis of 50 published female cases indicated a uniform lack of follow-up and significant morbidity—intellectual disability (10%), facial dysmorphism (28%), eye abnormalities (14%), and skeletal defects (28%).

**Conclusions:**

Our findings indicate normal ovarian function to date in a woman with an t(X;Y)(p22.3;q11.2). However, additional published studies in the literature suggest careful follow-up is necessary and contradict the generalization that females with Xp;Yq translocations are usually normal except for short stature.

**Electronic supplementary material:**

The online version of this article (doi:10.1186/s13039-015-0112-0) contains supplementary material, which is available to authorized users.

## Background

Translocations involving the X and Y chromosome may affect reproductive function. They occur because of pairing of homologous sequences of the pseudoautosomal regions of Xp and Yq in paternal meiosis [1]. These translocations are relatively rare in humans, but when they occur, they usually involve cytogenetic breakpoints at Xp22 and Yq11 [2]. The phenotype of affected individuals varies depending upon the number and specific genes deleted from the X chromosome. Affected males may present as stillborns or liveborn males with multiple congenital anomalies depending upon the number of X chromosomal genes that are deleted [2]. In contrast, females with X;Y translocations are usually reported to be short with normal intelligence and normal reproductive function, but some may have mild intellectual disability [2].

More than 50 X;Y translocation patients with breakpoints at Xp22 and Yq11 have been reported [2-20], but there are still many uncertainties regarding the specific molecular breakpoints involved, the phenotype of males and females, their long-term reproductive potential, and the risk of gonadal tumor formation. An extensive review of the literature indicates that of the reported female cases, follow-up was only rarely reported and that the phenotype is perhaps more severe than often stated [2-20]. However, there is no comprehensive analysis of the existing female cases to determine the prevalence of the reproductive and nonreproductive phenotypes. We present the clinical and molecular findings in a female proband and her family with an unbalanced X;Y translocation ascertained when her stillborn male was karyotyped. Taking into account this case and a critical evaluation of the literature, the purpose of the present study was to determine: 1) the precise molecular breakpoints of Xp22.31 and Yq11.2; 2) the phenotype of affected males and females; 3) ovarian function and fertility over time in the affected adult female; 4) the risk for cardiac anomalies; 5) the potential for a gonadal tumor in affected females and 6) comparison of this proband with all t(X;Y)(p22)(q11) females reported in the literature to understand the severity of the phenotype for genetic counseling purposes.

### Case presentation

**Figure 1:**
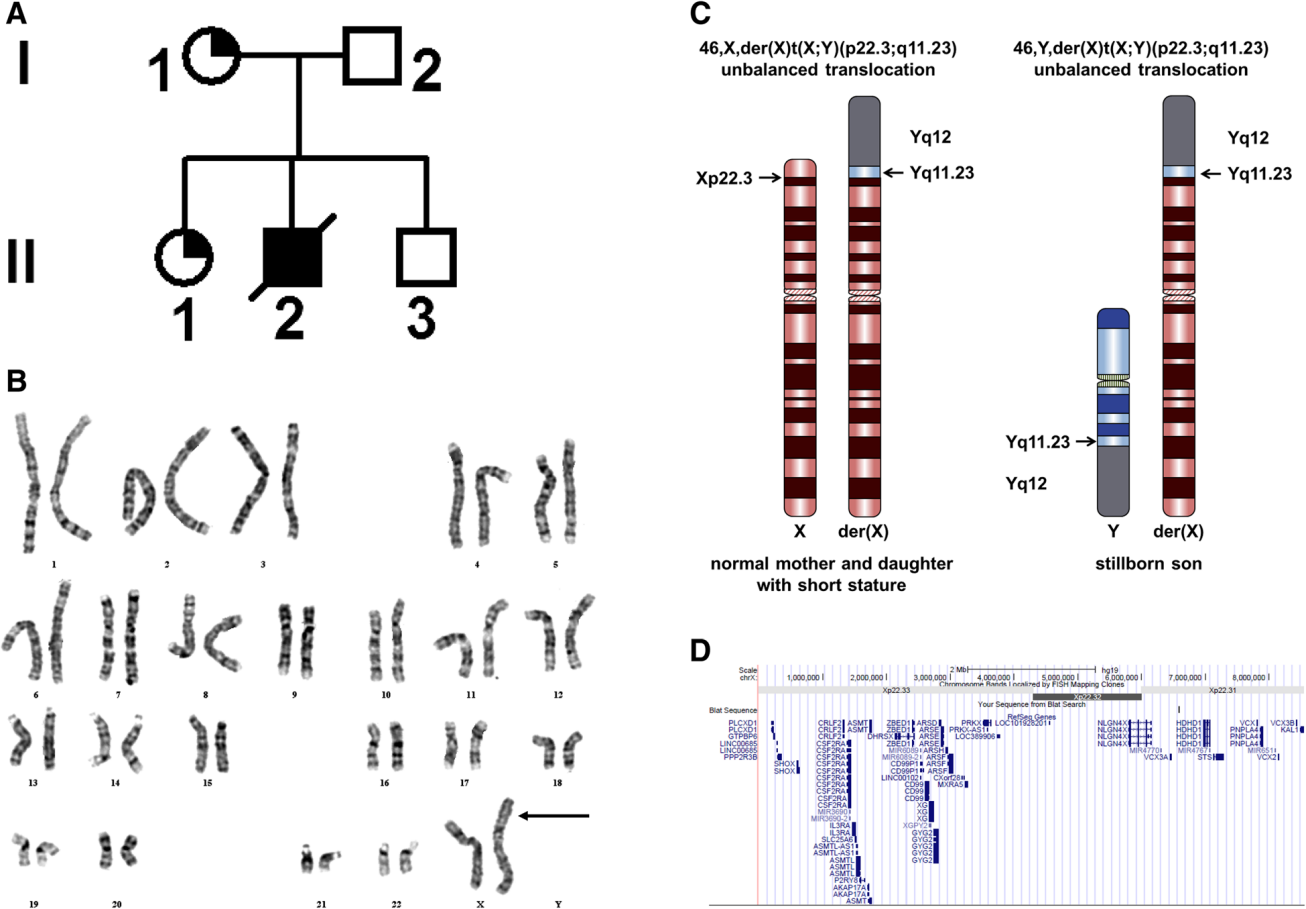
**The pedigree, karyotype, and genetic map are shown for the patient with the X;Y translocation. (A)** The pedigree of the family with unbalanced X;Y chromosome translocations is shown. Quarter shaded circles indicate females with the der(X) who have short stature. II-2 is a stillborn male with the der(X) chromosome. **(B)** Karyotype of t(X;Y)(p22;Yq11) is shown. The der(X) is indicated by an arrow. **(C)** The ideogram of the karyotype in the female and corresponding karyotype in the stillborn male. Y chromosome sequences are shown in gray shading. **(D)** Genetic map documents the 44 genes deleted from this der(X) chromosome. Sequencing of the junction fragment shows that the breakpoint lies within the *KAL1* gene in Xp22.3.

At presentation, the proband was a 25-year-old white female G2P1011 with regular menses, who conceived spontaneously without medical intervention (Patient I1 in Figure 1A). Her physical exam was normal except for short stature (4′9″). Her first pregnancy resulted in the birth of a normal, healthy girl (II1) with short stature (<2.5%), but her second pregnancy ended at 20 weeks with the birth of a stillborn male (II2) with hydrocephalus (Figure 1A) who did not have a full autopsy. Of interest, upon second trimester analyte screening, her serum unconjugated estriol was undetectable. Karyotype of the stillborn male II2 revealed 46,Y,der(X)t(X;Y)(p22.3;q11.2). Fluorescent in situ hybridization (FISH) demonstrated ish der(X)t(X;Y)(wcpY+,wcpX+), with the presence of the *KAL1* (Kallmann syndrome-1) region and deletion of the *STS* (steroid sulfatase) locus, narrowing the breakpoint region on Xp22.

## Results

The female proband I1 had a complete history and physical exam. Ovarian reserve was assessed using enzyme immunoassays for cycle day #2 serum follicle stimulating hormone (FSH) and random antimullerian hormone (AMH). Since she later presented with infertility, a hysterosalpingogram and preconceptional labs were performed, as was a semen analysis on her male partner.

Both the proband I1 and her prepubertal daughter II1 had an identical unbalanced translocation 46,X,der(X)t(X;Y)(p22.3;q11.23) with the derivative X being deleted of Xp22.3 to the terminus and containing Yq11.2 to the telomere cytogenetically (Figure 1B and C). Interphase FISH studies on the proband’s peripheral lymphocytes demonstrated the presence of the X centromere (DXZ1+) and Yq12 (DYZ1+), but not the Y centromere (DYZ3-), indicating the presence of only Yq11q12 on the derivative (X) chromosome (Figure 1D). SNP arrays revealed that the breakpoint resided within intron 6 of the *KAL1* gene (NM_000216.2), indicating that this gene was disrupted and that 44 genes in distal Xp22.3 were deleted (Figure 1E). The junction fragment of the der(X) chromosome was amplified using three sets of primers that could only amplify the X and Y sequences, and these were of the predicted sizes. Nested PCR products generated the expected sized bands (Additional file 1: Figure S1A). These PCR products were then purified and sequenced, and both X and Y-specific sequences were identified. The breakpoint on Xp22.31 is between coordinates of 8,548,685 and 8,548,812 (within 128 bp) and that on Yq11.2 is between coordinates of 15,989,009 and 15,989,287 (within 288 bp) using hg19 of the Human Genome Browser.

However, because of extremely high homology of repeated sequences within these regions of Xp22 and Yq11, the precise breakpoint was not able to be ascertained. Sequenced tagged sites of the Y chromosome indicated the presence of Yq11 sequences in this affected female (Additional file 1: Figure S1B). No centromeric sequences or SRY were identified.

At age 25, proband I1 had a cycle day #2 FSH = 5.9mIU/m L (2.8-11.3, follicular phase) supporting normal ovarian reserve [21]. Because of her extreme short stature and the terminal Xp deletion, she had a cardiac echo to assess her cardiac function and aortic diameter, which was normal. Genetic counseling was provided, which included the 50% risk that males would inherit the der(X) chromosome and likely be stillborn and the 50% risk that female offspring would inherit the translocation and have short stature. The couple elected to attempt conception without intervention, and had a healthy, normal male II3 (presumably 46,XY—but they declined a karyotype).

**Figure 2:**
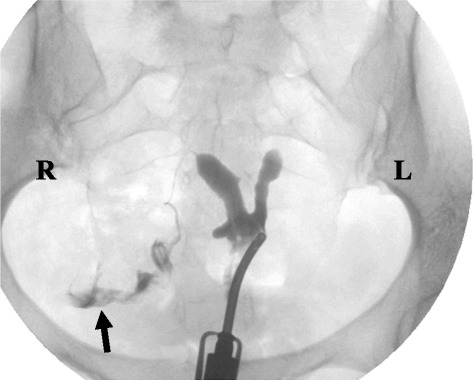
**Hysterosalpingogram showing a bicornuate vs. septate uterus with a patent right tube indicated by free spill of contrast (indicated by an arrow).**

The proband I1 maintained regular menses, but at age 32, she presented with three years of infertility. Her menses remained regular at 28–29 day intervals; and her physical exam including cardiac and reproductive systems, was normal. Her daughter (II1) remained below the 3% for height and was being evaluated for attention deficit disorder. The parents stated that the daughter was currently receiving one-on-one help in school due to difficulties in reading. During this one year follow-up period, the proband had a cycle day #2 FSH = 2.5mIU (2.8-11.3, follicular phase) and AMH = 5.2 ng/mL (1.1-6.9), two widely utilized tests of ovarian reserve [21]. Cystic fibrosis carrier testing, rubella immunity, thyroid studies, and prolactin were normal. A hysterosalpingogram revealed a bicornuate vs. septate uterus, a patent right tube, and no fill of the left tube (Figure 2). An ultrasound on cycle day 6 showed a normal uterus measuring 5.3 × 4.2 × 2.7 cm with a 3.3 mm (normal) endometrium and normal ovaries—right: 2.8 × 1.9 × 2.4 cm with a 7 mm follicle and 5 other smaller follicles; left: 2.2 × 1.4 × 2.5 cm with 3 small follicles. It was suggested she have ovarian tumor markers—inhibin A, inhibin B, hCG, AFP, and LDH, but these were declined.

She underwent clomiphene/intrauterine insemination (IUI) for four cycles with a 50 mg dose when there was a follicle on the right. Her estradiols were at appropriate preovulatory levels and ranged from 558–1395 pg/mL on cycle day 10–14 during monitoring (Additional file 1: Table S1) with at least one mature follicle. A serum progesterone performed 8 days after the LH surge was ovulatory. Total motile sperm ranged from 9–11 million for IUIs done on cycle days 12–17. She did not conceive after four cycles, but she continues to have regular menses.

## Discussion

Translocations involving the X and Y chromosomes are uncommon, although more than 50 Xp;Yq translocation cases have been reported worldwide [2,22]. In X;Y translocations, the breakpoints typically involve Xp22 and Yq11, which tend to result from pairing of homologous sequences located within these regions [2,22]. This was true in our translocation case, in which the sequenced portions of the der(X) chromosome contained both long interspersed nuclear elements (LINE/L1 and Line/L2 homologous sequences), enabling the exchange of genetic material during male meiosis. We have mapped the breakpoint of our patient with a 46,X,der(X)(t(X;Y)(p22.3;q11.21) translocation using FISH and SNP arrays, and cloned the breakpoint by long-range PCR and DNA sequencing.

The phenotype of females with Xp22;Yq11 translocations is usually stated to be normal except for short stature [2,22]. However, there has been no comprehensive analysis of published female cases, as most reports only contain one or several affected females; and follow up has only been reported for several patients. Upon an extensive literature review, at least 50 females with Xp22;Yq11 translocations have been reported (Table 1; Summary in Table 2). Critical review of these mostly isolated female cases indicates that there is considerably more phenotypic morbidity in many affected phenotypic females than is generally thought (Tables 1 and 2) [2-20]. We realize that there may be a certain degree of publication bias in that more severe phenotypes may be present in the literature. Nevertheless, this is the information now available to evaluate the phenotypic effects of this translocation.

**Table 1:** **Reported Females with Xp22;Yq11 Translocations**

**No.**	**Reference**	**Index Case**	**Age at dx**	**Age at F/U**	**Menarche**	**Ovarian function reported (Fertility)**	**Stature**	**Endocrine Studies**	**AMH**	**Menopause**	**Other abnormalities**
1	**Khudr 1973** [3]	Female 46,X,t(X;Y)(p22;q11)	31	Not followed	12 yr	4 SABs; 1 daughter (G5P1041); regular menses	Short, 137 cm	FSH = 9.1mIU/ml; Normal LH & thyroid studies	Not measured	n/a	exploratory laparotomy revealed nl uterus, tubes, and cystic ovaries;
2	**Van den Berghe 1977 (1)** [4]	Female 46,X,t(Xp;Yq)	14	17 yr	14 yr	n/a	Short, 145 cm at 14yo; 150 cm at 17 yr	FSH = 5.8 mU/ml	Not measured	n/a	Bicornuate/slightly asymmetric with left horn less developed and filiform, fibrous tubes; nl appearing ovaries
3	**Van den Berghe 1977 (2)** [4]	Female 46,X,t(Xp;Yq)	37	Not followed	Unknown	G4; macerated normal looking male 8 mo; 1 full term female; 1 ?abnl male newborn (short) ; 1 nl male	Not reported	Not measured	Not measured	n/a	None reported or evaluated
4	**Van den Berghe 1977 (3)** [4]	Daughter of (2) 46,X, t(Xp;Yq)	2	Not followed	n/a	n/a	Short, birth 43 cm; 3rd percentile at 2 yr	Not measured	Not measured	n/a	None reported or evaluated
5	**Akesson** [5]	Swedish woman with 46,X,der(X),t(X;Y) (p22;ql1)	Not stated	n/a	Not given	Two affected male sons with ID & anomalies	150 cm	Not measured	Not measured	n/a	
6	**Johnston 1987** [7]	Female 46,X,t(X;Y)(p22;q11)	birth	7.5mo	n/a	Primordial follicles on ovarian bx	48.5 cm at 3 days old; 60.5 cm at 7.5 mo	FSH = 8.5 ng/mL at 12 days old; “Normal” at 7.5 mo	Not measured	n/a	Normal uterus by u/s and visual exam during surgery; imperforate anus, mesomelic dwarfism
7	**Pfeiffer 1980** [6]	Female 46,X,t(X;Y)(p22;q11)	34	Not followed	14yo	G2; 1SAB; 1 abnl male	Short, 144.5 cm	Not measured	Not measured	n/a	Microcephaly, borderline ID
8	**Agematsu 1988** [8]	Female 46,X,+der(X), t(X;Y)(p22.3q12);parents NA	28	30 yr	Unknown	Normal breasts; Apparent fertility; G1P1001; abnl son	151 cm	Not measured	Not measured	Unknown	Mildly short upper extremities; No punctate calcification by X-R
9	**Al-Gazali 1990** [9]	De novo 46,X,t(X;Y)(p22;q11)	birth	8mo	n/a	Not determined	<5% at 8 mo	Not measured	Not measured	n/a	Linear skin defects with scalded appearance of upper neck & face, severe bilateral microphthalmia, left corneal opacity, syndactyly of 2nd/3rd toes of left foot, normal early developmental milestones
10	**Al-Gazali 1990** [9]	De novo 46,X,t(X;Y)(p22;q11)	birth	2 yr 10mo	n/a	Not determined	<5% at 2 yr 10 mo	Not measured	Not measured	n/a	Bilateral microphthalmia, left orbital cyst, linear skin defects on face, neck, shoulder, & chest; anteriorly displaced anus; 1 cm sacral nevus, lack of sphincter control of bowel & bladder at 2 10/12 yr, problems walking long distances, otherwise normal developmental milestones
11	**Kuznetzova 1994** [11]	Female 46,X,t(X;Y)(p22.3;q11)	12	14 yr	Unknown, but “normal” at 13 yr; normal breasts	n/a	Short	Normal FSH, LH, Estradiol; low Progesterone	Not measured	n/a	Hypoplastic uterus, short tubes, numerous follicles within ovaries; short neck; mild pectus excavatum;
12 to 32	**Hsu 1994** [10]	25 females 46,X,+der(X),t(X;Y)(p22;q11); includes cases 1–4; 6	Unknown	Not followed	Unknown	“Proven fertility” or said to have nl ovaries in 20 of 25	17 of 22 with height info reported as short	Not measured	Not measured	Unknown	None reported or evaluated
33	**Joseph 1996 (1)** [13]	AA Female de novo 46,X,der(X)t(X;Y)(p22;q11)	prenatal	4 yr	n/a	n/a	Short, 71 cm (<5%) at 2 yr treated with growth hormone	At 23 mo, FSH = 3.0 mIU/ml, GnRH stimulation test normal; low GH	Not measured	n/a	Premature thelarche (T3 breasts); achieved 30% with GH at age 4
34 & 35	**Joseph 1996 (2)** [13]	Caucasion twin females with de novo 46,X,der(X)t(X;Y)(p22.3;q11.21)	prenatal	2 yr 10mo	n/a	n/a	Short, 81.5 cm and 81 cm at 2 yr (both <5%)	IGF-1 and IGFBP3 normal	Not measured	n/a	Dolichocephaly, Narrow flat face, downslanting palpebral fissures, & epicanthal folds
36	**Joseph 1996 (3)** [13]	White Female de novo 46,X,der(X)t(X;Y)(p22.3;q11)	prenatal	22mo	n/a	n/a	Short, 53.3 cm at birth and fell to 5th percentile at 22mo	IGF-1 and IGFBP3 normal	Not measured	n/a	None reported or evaluated
37	**James 1998 (1)** [12]	Female 46,t(X;Y)(p22.31;q11.21)	24.25	Not followed	Unknown	No evidence of ovarian failure	Short	Not reported	Not measured	n/a	Upslanting palpebral fissures, increased carrying angle, high arched palate
38	**James 1998 (2)** [12]	Female 46,X,t(X;Y)(p22.33;q12)	5.8	Not followed	n/a	n/a	Short	Not measured	Not measured	n/a	Convergent strabismus, edema
39	**James 1998 (3)** [12]	Female, mom of (2), 46,X,t(X;Y)(p22.33;q12)	48.9	Not followed	Unknown	No evidence of ovarian failure	Short	Not measured	Not measured	Unknown	Low posterior hairline, short, distal phalanges of thumbs, renal anomaly, schizoid disorder
40	**James 1998(4)** [12]	Female 46,X,der(X)t(X;Y)(p22.3;q11.2)	Unknown	Not followed	Unknown	n/a	Short	Not measured	Not measured	Unknown	Microphthalmia, linear skin defect
41	**Guichet 1997** [17]	Female with de novo 46,X,t(X;Y)(p22.33;q11.23)	20	23 yr	14 yr	Unknown and stated that breasts developed after estrogen and progesterone treatment	Short, 146 cm	Not measured	Not measured	n/a	Madelung deformity; Small hands with moderate camptodactyly of 2-5th digits of hands; short feet with shortening of all toes and moderate syndactyly 2-5th toes; needed speech therapy
42	**Calabrese 1999** [15]	Female with 46,X,der(X)t(X;Y)(p22.3;q11.21)	34	NA	NA	Ascertained through a son with LWD	150 cm	Not measured	Not measured	n/a	Bowing of radius & bilateral subluxation of distal ulna (Dx with Leri-Weill dyschondrosteosis)
43	**Frints 2001** [16]	Female with 46,X,der(X)t(X;Y)(p22.3;q11.21)	Reported when son 1yo, mother “young”	Not followed	Unknown	G6; 3 male SBs, 2 healthy daughters, 1 abnormal term male	Normal, 161.3 cm (not short)	Not measured	Not measured	n/a	height 25-50%
44	**Speevak 2001** [2]	Female with de novo 46,X,der(X)t(X;Y)(p22.3;q11.2)	prenatal	3 ¾ yr	n/a	n/a	Short, 50 cm at 6 weeks, below 3rd percentile at 3 ¾ years	Not measured	Not measured	n/a	None reported or evaluated
45	**Bukvic 2010 (1)** [14]	Female with 46,X,der(X),t(X;Y)(p22;q12)	20 m	Not followed	n/a	n/a	Short, 77 cm	Not measured	Not measured	n/a	Brachycephaly, mesomelia, mild developmental delay (IQ = 83), global motor delay, major linguistic deficits, mild facial dysmorphic features
46	**Bukvic 2010 (2)** [14]	Female with 46,X,der(X),t(X;Y)(p22;q12), mother of Bukvic case 1 (#42)	27	Not followed	Unknown	G6; 1 abnl male, 1 abnl female, 4 SABs within 10 weeks	159 cm	Not measured	Not measured	n/a	Mesomelia; Madelung deformity; normal IQ
47	**Chen 2012** [18]	Female de novo with 46,X,der(X)t(X;Y)(p22.31;q11.221)	prenatal- 17 weeks	Termination of pregnancy 21 weeks	Unknown	n/a	25 cm at 21 weeks	Not measured	Not measured	n/a	Fetal demise, Shortening of humerus and femur
48	**Palka-Bayard-de-Volo 2012** [20]	Female de novo with 46,X,der(X)t(X;Y)(p22;q11)	11y9mo	Not followed	n/a	n/a	Short, 44 cm at birth, −3.78 SD at eval (<5%)	Normal thyroid, IGF-1, & celiac studies; normal brain MRI	Not measured	n/a	Facial dysmorphism (hypertelorism, epicanthus, short philtrim, and simple external ear; ASD, psychomotor delay, major language impairment, mild ID (IQ = 70)
49	**Cheng 2013 (1)** [19]	Female with 46,X,der(X)t(X;Y)(p22;q11.2)	Unknown	Not followed	Unknown	G1, P1; abnormal male	150 cm	Not measured	Not measured	n/a	None reported or evaluated
50	**Cheng 2013 (2)** [19]	Female with 46,X,der(X)t(X;Y)(p22;q11.2)	Unknown	Not followed	“Normal”	2y hx infertility due to tubal disease; successful IVF; 2 previous TABs	Short, 1.51 m	Normal FSH, LH, Prolactin, 17-OH Preg, estradiol, & progesterone (values not provided)	Not measured	n/a	HSG showed blocked tubes; no uterine abnl reported

**Table 2:** **Clinical characteristics of phenotypic females with Xp22;Yq11 translocations**

**Phenotype**	**Total (percent)**		**Details**
	**All Ages**	**Reproductive age**	
**Reproductive**			
Fertility	9/29 (31%)	9/10 (90%)	
Delivery of normal child	4/29 (13.8%)	4/10 (40%)	4 of 10 adults had a normal child
Uterine anomaly	2/29 (6.9%)		Hypoplastic (n = 1); bicornuate (n = 1)
FSH Levels	6/29 (20.7%)	4/10 (40%)	All normal, but only 4 of reproductive age & none were done on cycle day 2-3
AMH Levels	0		None were reported
Follow up	11/29 (37.9%)	3/10 (30%)	No reports of repeat FSH or fertility evaluation
Gonadoblastoma	0		
**Nonreproductive**			
Short stature	27/29 (93.1%)		
Intellectual disability	3/29 (10.3%)		Microcephaly/borderline ID (n = 1);Major language delay/mild ID with IQ =70 (n = 1); Mild developmental delay, IQ = 83, global motor delay, major linguistic deficits (n = 1)
Facial dysmorphism	8/29 (27.6%)		Linear skin defects of face (n = 3); upslanting palpebral fissures, brachycephaly, others
Eye abnormalities	4/29 (13.8%)		Microphthalmia (n = 3); strabismus (n =1)
Cardiac defects	1/29 (3.4%)		ASD
Skeletal defects	8/29 (27.6%)		Madelung, mesomelia, camptodactyly, syndactyly, others

Only included in this table are patients 1–11 and 33–50 since the details from Hsu (cases 9–29) are not provided. There are a total of 29 patients (all ages) and 11 that are reproductive age (defined as ≥17 years). ID = intellectual disability, ASD = atrial-septal defect. For full details including references, see Table 1.

While it is true that more than 90% of affected females have short stature, other significant phenotypic findings are commonly present and include mild intellectual disability (10%), facial dysmorphism (28%), eye abnormalities (14%), and skeletal defects (28%). One of 29 (3%) females had a congenital heart defect [20]. In cases where the female has been characterized, fertility has only been reported in 10 translocation patients and nine conceived; however only 4/10 (40%) had a normal child (Tables 1 and 2). Serum FSH to assess ovarian reserve has been performed (and is normal) in ~20% (6/29) of affected females, but was only reported in four postpubertal females—the time when it has clinical significance [23]. In the childhood period, FSH does not accurately reflect ovarian function, as gonadal failure cannot be differentiated from normal ovarian function [23]. Only three reproductive aged Xp22;Yq11 females have had short-term follow up, and no patient has had a cycle day 2–3 FSH (cycle day is not specified) or a random serum AMH level analyzed for ovarian reserve [21]. Therefore, ovarian reserve has not ever been adequately assessed in these previously reported women. Interestingly, in all published cases, we could not find any reports of a Xp22;Yq11 translocation female with a gonadoblastoma.

Our proband I1 is a normal female except for short stature, who has demonstrated normal reproductive function as evidenced by regular menses. Her ovarian reserve is normal with a normal cycle day #2 FSH at age 25; and normal day #2 FSH and AMH at age 32. She also had normal ovulatory response to superovulation with clomiphene. Her first child was a daughter (II1) with the same unbalanced translocation, who is also normal except for extreme short stature and attention deficit disorder. She has not yet had breast development at age 9. Short stature in these two females is at least partially explained by the heterozygous loss of the *SHOX* on the distal Xp22.3, which is thought to be involved in the short stature of Turner syndrome [24]. Patients with monosomy X (with or without mosaicism) have a 50% risk of congenital cardiac anomalies [25], so this was a concern in our patient who had monosomy for a large telomeric region of Xp. However, she does not have any murmurs and had a normal echocardiogram. She has been without any cardiac symptoms during her 7 year period of follow-up. These findings, as well as our identification of only one of 29 (3%) translocation patients having a cardiac anomaly (an atrioseptal defect) in the literature [20], suggest that the genes in this deleted region do not play a major role in the heart defects of Turner syndrome. However, it is also possible that our patient could develop aortic valve abnormalities and/or a dilated aortic root in the future, so careful follow up is recommended.

Our proband’s second pregnancy resulted in a stillborn male II2 with hydrocephalus, who inherited the derivative X chromosome from his mother. Since approximately 9.5 Mb of distal Xp containing 44 genes is deleted in this male, this likely resulted in embryonic lethality. Many of these genes are recognized to be involved in human genetic diseases and include: *SHOX* (dyschondrosteosis or Leri-Weill syndrome) [24], *NLGN4* (autism and intellectual disability) [26], *ARSE* (chrondrodysplasia punctata, which has skeletal abnormalities and microcephaly) [27], *VCX3A* (implicated in intellectual disability) [28], and *STS* (ichthyosis) [29]. Since *KAL1* is directly interrupted, it is likely this male would also have had Kallmann syndrome had he survived [30,31]. However, since this is an X-linked recessive disorder not affecting women, it is not expected that his mother would be affected with hypogonadotropic hypogonadism or anosmia (and she was not).

This couple received genetic counseling, including the possibility of preimplantation genetic diagnosis, which they declined. Fortunately, our proband had a normal term delivery of a reportedly normal male II3 in a subsequent pregnancy. The segregation of the der(X) within the family is what would be predicted. Since the mother is heterozygous for the der(X), she has a 50:50 chance of transmitting this to female offspring so that half of females would be expected to be short with other possible anomalies and the other half normal. In contrast, she has a 50:50 chance of passing the der(X) to a male which would result in deletion of 44 genes and probable lethality. If a male received her normal X chromosome, he would be expected to be normal. Although not karyotyped, her normal son is presumably 46,XY.

The presence of Yq11q12 sequences on der(X) in the female proband is unlikely to affect the phenotype. Since *SRY* (sex determining region on the Y chromosome) is localized to the distal Yp arm, its absence did not interfere with female sexual development. One to two megabase sequences in the pericentromeric region of Y, which may contain *GBY* (gonadoblastoma locus on the Y chromosome), are thought to contribute to the risk of gonadoblastomas and subsequent germ cell neoplasms of the ovaries in females with a Y chromosome [32]. Our patient has no evidence for this phenotype, and this is understandable given that the breakpoint at Yq11.21, which when revised after breakpoint cloning, is about 3.5 Mb away from this centromeric region. It is also possible that the *GBY* locus is in the pericentromeric region of Yp, which is not present in our patient. TSPY, another putative gonadoblastoma locus, is also localized to Yp and should not be present in this female proband. It is also reassuring that we were unable to document a female with t(X;Y)(p22;q11) with a gonadoblastoma (Tables 1 and 2). However, caution must be exercised since many of these patients had no published follow up, and follow up with tumor markers could be considered in her long term care.

The sequences from Yq11 include putative spermatogenesis genes documenting the presence of euchromatin on the der(X), but they are unlikely to be of any clinical significance in females. Our patient also had a uterine anomaly. Uterine anomalies were present in two of 29 cases found in the literature—one hypoplastic [11] and one bicornuate [4](Tables 1 and 2). However, it is unknown whether or not the translocation contributed to the (likely) bicornuate uterus in our patient.

## Conclusion

In conclusion, mapping the breakpoint and cloning the junction fragment of the der(X) chromosome in our patient with a Xp22;Yq11 translocation resulted in the identification of a 9.5 Mb deletion of distal Xp22, which contains 44 genes. Our findings suggest that this der(X) in affected females: 1) does not affect regular menstruation or ovarian reserve; 2) does not preclude fertility; 3) results in short stature; 4) does not result in gonadoblastomas in females by age 32; 5) does not result in structural cardiac disease in females like 45,X individuals; and 6) results in the loss of 44 genes that collectively are probably lethal in affected males. Additionally, significant morbidity in the form of intellectual disability (10%), facial dysmorphism (28%), eye abnormalities (14%), and skeletal defects (28%) may occur in affected females, which should be considered in the genetic counseling of these patients.

## Methods

When the patient was seen at Georgia Regents University, karyotypes were performed on the proband I1 and her healthy daughter II1 using standard G banding methods from peripheral white blood cells (WBCs) [33]. She signed a consent to have her records reviewed and to have blood drawn for molecular studies, which were approved by the Human Assurance Committee of Georgia Regents University. A lymphoblastoid cell line was created by infecting WBCs with Epstein-Barr virus and using cyclosporine A as described previously [34]. FISH was performed on metaphase chromosomes using standard methods with five fluorescently labeled probes including: X centromere (DXZ1), Y centromere (DYZ3), Yq12 (DYZ1), *STS*, and *KAL1* genes. A Genome-Wide Human SNP Array 6.0, which features 1.8 million genetic markers, including more than 906,600 single nucleotide polymorphisms (SNPs) and more than 946,000 probes, was used to exclude copy number variants (CNV) and narrow the breakpoint. The Affymetrix GeneChip Command Console (AGCC) was used to scan the chips and the Genotype Console 2.0 was used to analyze the data to generate microarray results.

Long–range polymerase chain reaction (PCR) was performed to amplify the junction fragment of the derivative X chromosome. Primers were designed to amplify only fragments that contained both X and Y sequences (Table 3). Primers were Y-2040for and X-4177rev (to yield a 9 kb fragment), Y-6440for and X-2150rev (to yield a 4 kb fragment), and Y-4140for and X-5891rev (to yield a 10 kb fragment). PCR conditions included a 93°C denaturation step for 3 minutes, followed by 35 cycles of 93°C for 15 seconds, 62°C for 30 seconds, and 68°C for 10 minutes. PCR products were then electrophoresed on 1.2% agarose gels in the presence of a molecular weight marker, stained with ethidium bromide, and photographed. Nested PCR using internal primers was then used to amplify the junction fragment of the breakpoint region. The resulting PCR products were then ethanol precipitated and sequenced using the dideoxy method with the Big Dye Terminator Kit and run on the ABI 310 Automated DNA Sequencer [35]. Multiple bioinformatic databases were utilized including Human Genome Browser (http://genome.ucsc.edu/), Repeat Masker Web Server (http://www.repeatmasker.org/cgi-bin/WEBRepeatMasker), ClustalW2 (http://www.ebi.ac.uk/Tools/clustalw2/index.html) and WWW READSEQ Sequence Conversion (http://www-bimas.cit.nih.gov/molbio/readseq).

**Table 3:** **Primers used to amplify the junction fragments**

**Primer**	**Sequence (5′ → 3′)**
Xp22.31-2150rev	AGATGTGGCAGCATCTTGTTAGTGTACTGGTTAAGTC
Xp22.31-4177rev	CACATTGCATATGTCTCATTGTGAGGAGCATCC
Xp22.31-5891rev	GTGACATGTTCCCTGTGCTCTGTGACATGTC
Yq11.2-2040for	AGTAAGGATCTTTCGACATTTGGTGAGAATGAGAAACAGA
Yq11.2-4140for	GTCTGAAACCCAGGATCCGGAAGTGGG
Yq11.2-6440for	CTTACATAGGAATATGCAGACACATTAACACCTTGTGCTC

To determine which Yq11 sequences were contained in the der(X) chromosome, 19 sequence tagged sites (STS) on various regions were amplified according to the instructions of the Y Chromosome Deletion Detection System Version 2.0 (Promega; Madison, WI) and electrophoresed on 1.2% agarose gels.

## Additional file

Additional file 1: Figure S1. (A) Agarose gel electrophoresis showing successful amplification of the junction fragment (see Methods). These two PCR products were the expected size using X forward primer and Y reverse primers (lanes 1 and 2). A molecular weight marker is shown in lane 3. (B) Sequence tagged sites of the Y chromosome indicate the presence of Yq11 sequences. F = female with der(X); M = control male; C = negative control, which contains all reagents except for DNA; L = 123 base pair ladder, which was used as a molecular marker. **Figure S2.** FISH analysis of metaphase chromosomes shows the derivative chromosome der(X) as indicated by the presence of centromeric DXZ1 and the absence of Y centromeric DYZ3. Also shown is the presence of *KAL1* on both of her X chromosomes and the absence of *STS* on one of the X chromosomes, suggesting that the breakpoint lies between the two genes. **Table S1.** Four clomiphene (50 mg/day cycle days 3–7)/intrauterine inseminations (IUI). Ovulation was documented in one cycle with a serum progesterone >20 ng/mL (>3 ng/mL is ovulatory). CD = cycle day.
