# Supplementary material for: Long-term follow-up of females with unbalanced X;Y translocations—reproductive and nonreproductive consequences
Source: Mol Cytogenet. 2015 Feb 22;8:13. doi: 10.1186/s13039-015-0112-0 (PMC4347569; doi:10.1186/s13039-015-0112-0)
Supplement: Additional file 1: Figure S1. — (A) Agarose gel electrophoresis showing successful amplification of the junction fragment (see Methods). These two PCR products were the expected size using X forward primer and Y reverse primers (lanes 1 and 2). A molecular weight marker is shown in lane 3. (B) Sequence tagged sites of the Y chromosome indicate the presence of Yq11 sequences. F = female with der(X); M = control male; C = negative control, which contains all reagents except for DNA; L = 123 base pair ladder, which was used as a molecular marker. Figure S2. FISH analysis of metaphase chromosomes shows the derivative chromosome der(X) as indicated by the presence of centromeric DXZ1 and the absence of Y centromeric DYZ3. Also shown is the presence of KAL1 on both of her X chromosomes and the absence of STS on one of the X chromosomes, suggesting that the breakpoint lies between the two genes. Table S1. Four clomiphene (50 mg/day cycle days 3–7)/intrauterine inseminations (IUI). Ovulation was documented in one cycle with a serum progesterone >20 ng/mL (>3 ng/mL is ovulatory). CD = cycle day. [file 13039_2015_112_MOESM1_ESM.pdf]

Supplemental Figure 1. (A) Agarose gel electrophoresis showing successful amplification of the junction fragment (see Methods). These two PCR products were the expected size using X forward primer and Y reverse primers (lanes 1 and 2). A molecular weight marker is shown in lane 3. (B) Sequence tagged sites of the Y chromosome indicate the presence of Yq11 sequences. F= female with der(X); M = control male; C= negative control, which contains all reagents except for DNA; L = 123 base pair ladder, which was used as a molecular marker.

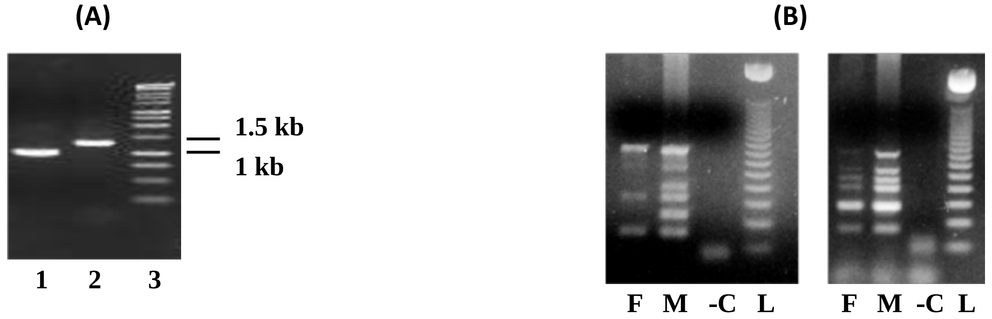

Supplemental Figure 2. FISH analysis of metaphase chromosomes shows the derivative chromosome der(X) as indicated by the presence of centromeric DXZ1 and the absence of Y centromeric DYZ3. Also shown is the presence of *KAL1* on both of her X chromosomes and the absence of *STS* on one of the X chromosomes, suggesting that the breakpoint lies between the two genes.

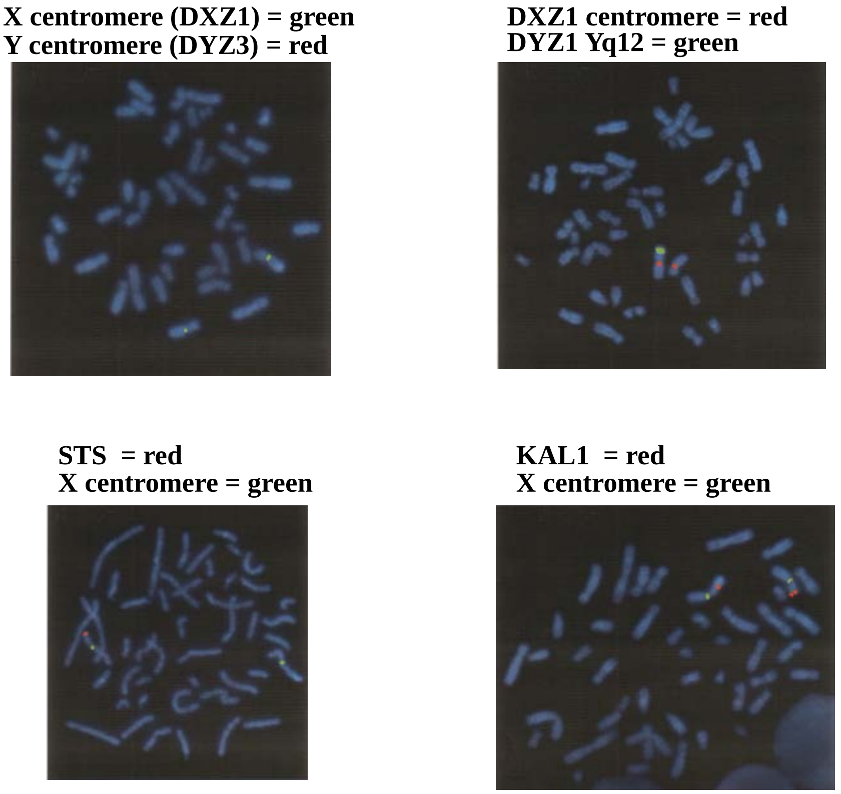

Supplemental Table 1. Four clomiphene (50mg/day cycle days 3-7)/intrauterine inseminations (IUI). Ovulation was documented in one cycle with a serum progesterone >20ng/mL (>3ng/mL is ovulatory). CD = cycle day.

Cycle	CD#	Follicles ≥10mm & size in mm	Estradiol (pg/mL)	LH (mIU/mL)	IUI	
					Total motile sperm in millions	CD#
Cycle #1	14	23.5, 10.4mm	558	10.4	11	16
Cycle #2	11	17,14,14,13.5	1395	Urine LH surge CD#12	10	14
Cycle #3	10	31, 18, 16, 14, 14	750	6.4	9	12
Cycle #4	11	13, 12, 12, 10	503	Urine LH surge CD#15	14	17
